# Philtral Columns and Nostril Shapes in Nigerian Children: A Morphometric and Aesthetic Analysis

**DOI:** 10.1155/2013/382754

**Published:** 2013-06-11

**Authors:** Ibrahim Abdulrasheed, Asuku Malachy Eneye

**Affiliations:** Division of Plastic Surgery, Department of Surgery, Ahmadu Bello University Teaching Hospital, P.O. Box 06, Shika, Kaduna State, Zaria 810001, Nigeria

## Abstract

*Background.* The upper lip-nose complex contributes significantly to the concept of symmetry and proportion of the face. A study of the morphology and aesthetic preferences of the lip-nose complex will provide a database that will serve as a guide for reconstruction. *Subjects and Methods.* Hundred Nigerian children participated in this study. Demographic data and standard photographs of the philtral column and nostrils were obtained. Sixty volunteers were recruited to evaluate the photographs. Each volunteer was asked to rank the photographs based on their aesthetic preference. *Results.* The morphology of the philtral columns was classified into four groups: (1) triangular, (2) concave, (3) flat, and (4) parallel. The nostril shape was also classified into four groups: (1) triangular, (2) round, (3) teardrop, and (4) rectangular. In both genders, the triangular shape of philtral column was the most common. There are significant age differences in the aesthetic rankings of philtral columns and nostril shapes. *Conclusion.* Our study establishes the basal values for the morphometric and aesthetic parameters of the lip-nose complex of 5- and 6-year-old children in Nigeria. We hope our results and reconstructive surgery will intersect at a point to treat disfigurements of the philtrum and nostrils successfully.

## 1. Introduction

The human face is one of the most attractive parts of the body [1]. It is central to many aspects of social interaction and the visual perception of the face is influenced by a complex combination of various factors such as appearance, expression, and symmetry. Earlier reports [2, 3] have shown that there is a proportional relationship between symmetry and attractiveness, and symmetrical faces are generally perceived as more attractive. 

The upper lip-nose complex is an important aesthetic facial unit. It contributes significantly to the concept of symmetry, harmony, and proportion of the face [1, 4]. The philtrum is the central unit of the upper lip and plays a key role in the appearance of the lip and nostril [5, 6]. It consists of the dimple, two philtral ridges, the tubercle, and the white roll between the two high points of Cupid's bow. The philtrum is especially prominent during conversation and facial expression [7]. During labial movement, a dimple is formed, with accentuation of the philtral ridges [5]. The nose is located in the middle of the face and is the most defining feature. Thus, it naturally attracts the gaze of the onlooker [4, 8]. The shape of the nostril is a signature indicating the ethnicity, race, age, and sex [9]. Given its importance and ability to change the appearance of the face, asymmetry of the nostrils will affect overall facial appearance [2, 10]. It is, however, also well recognized that disfigurements of the philtral columns and nostrils from congenital and acquired deformities causes significant emotional distress. This is because any deformity of the face has always been considered as one of the least desirable handicaps [3, 11]. 

Research provides evidence that the study of the morphology of the Lip-nose complex at various ages serves as a guideline for reconstruction [12–15]. Corresponding studies in Nigeria are very scarce. Furthermore, popular views of aesthetics of the face continue to evolve as our communities become more diverse and as the media and popular culture increasingly influence our tastes. A thorough understanding of current societal preferences will help guide surgical planning for aesthetic surgery of the face [16]. The purpose of this study is thus twofold: (1) to establish the morphology of philtral columns and nostril shape in Nigerian children, and (2) to identify the aesthetic preferences of philtral column and nostril shape in Nigerian children. 

## 2. Subjects and Method

### 2.1. Morphology of Philtral Column and Nostrils

**Figure 1:**
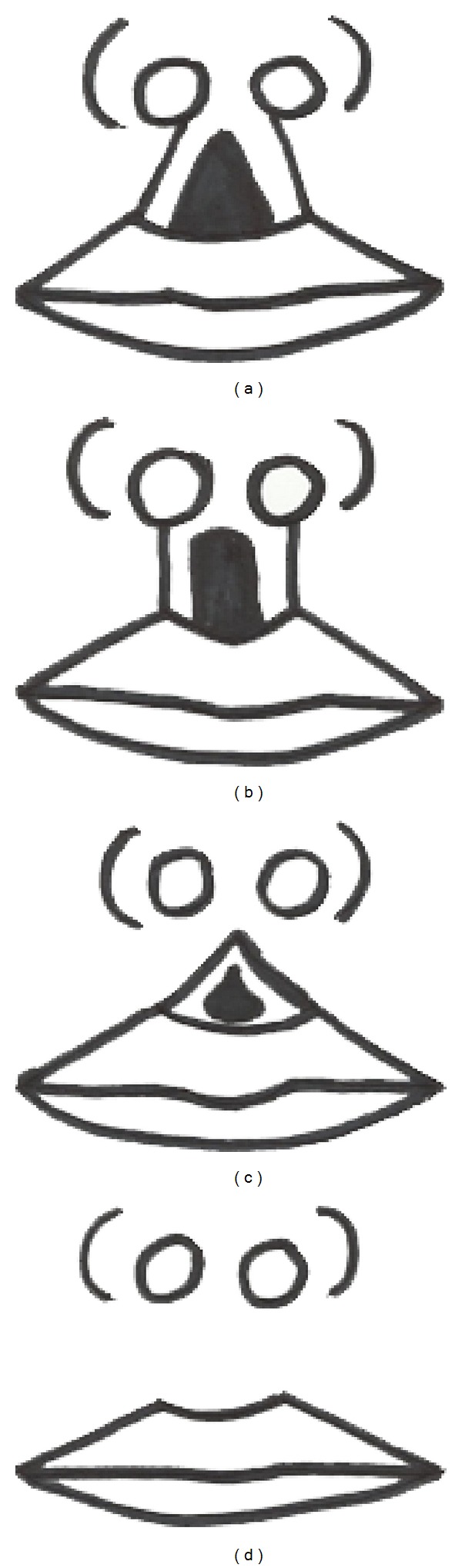
Diagram of philtral shape classification, Mori et al. [13]. (a) Triangular type, (b) parallel type, (c) concave type, and (d) flat type.

**Figure 2:**
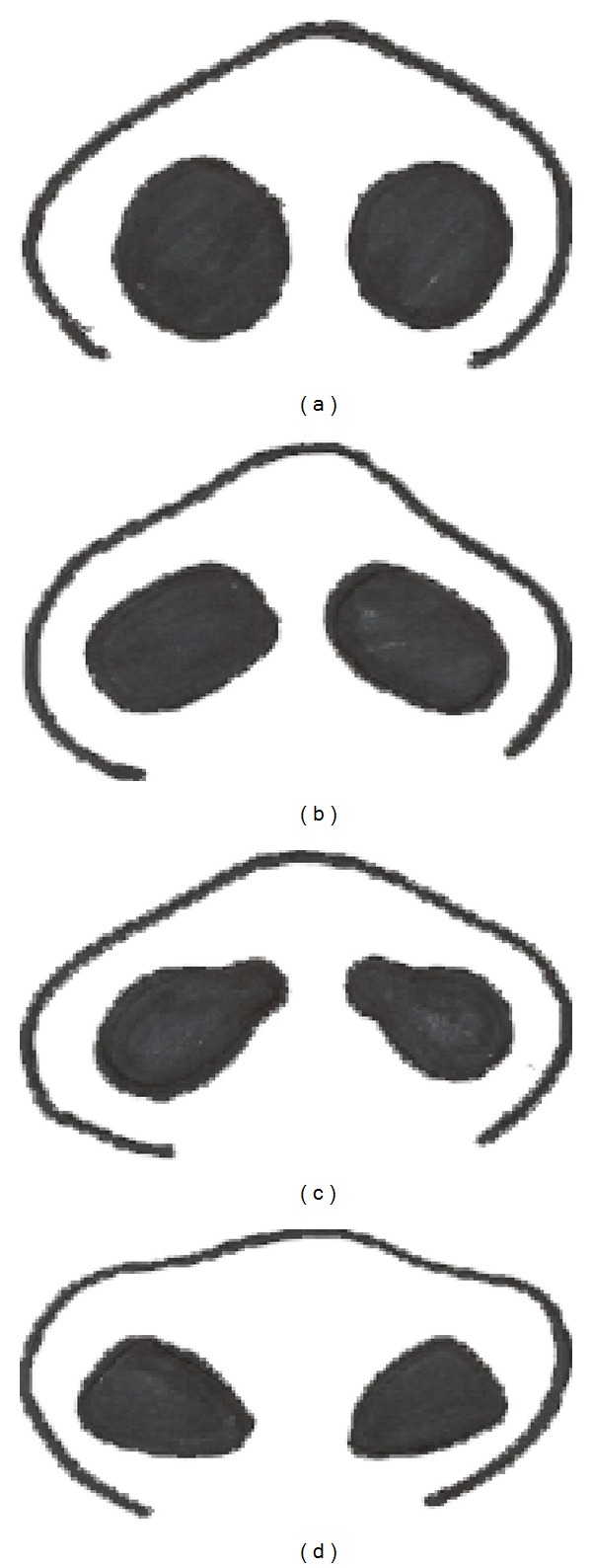
Diagram of nostril shape classification. (a) Round type, (b) rectangular type, (c) teardrop type, and (d) Triangular type.

Subjects included in this study were required to be 5 or 6 years of age and both parents to be of Nigerian heritage. There were 100 Nigerian children (54 males, 46 females). The boys were aged 6 years (24 children) and 5 years (30 children) and the girls were aged 6 years (22 children) and 5 years (24 children). Other inclusion criteria included no history of craniofacial syndromes, major trauma, or previous plastic and reconstructive surgery of the face. We analyzed the shape of the philtral columns and nostrils and classified it into four groups. These groups are similar to those described by Mori et al. [13] Figure 1.  Figure 1(a) triangular type: the origins of the philtral column are located near both sides of the medial crural footplates. The philtral dimple is approximately triangular.  Figure 1(b) parallel type: the philtral columns originate from the nostril sills and exhibit an almost parallel shape with a prominent dimple along the upper lip.  Figure 1(c) concave type: the philtral columns begin in the lower half of the upper lip and the dimple is emphasized. There is no philtrum dimple in the upper half of the upper lip.  Figure 1(d) flat type: the philtral columns have almost no prominence with a vaguely delineated dimple. The nostril shapes were classified into four groups: Figure 2(a) round type, Figure 2(b) rectangular type, Figure 2(c) teardrop type, and Figure 2(d) triangular type (Figure 2). 

Statistical evaluation of data was performed on SPSS 17.0 (SPSS Inc., Chicago, IL) statistical package program for Windows. Age and sex differences were evaluated using *t*-test and *P* < 0.05 was considered to be statistically significant.

### 2.2. Aesthetic Preferences of Philtral Column and Nostril Shape

**Figure 3:**
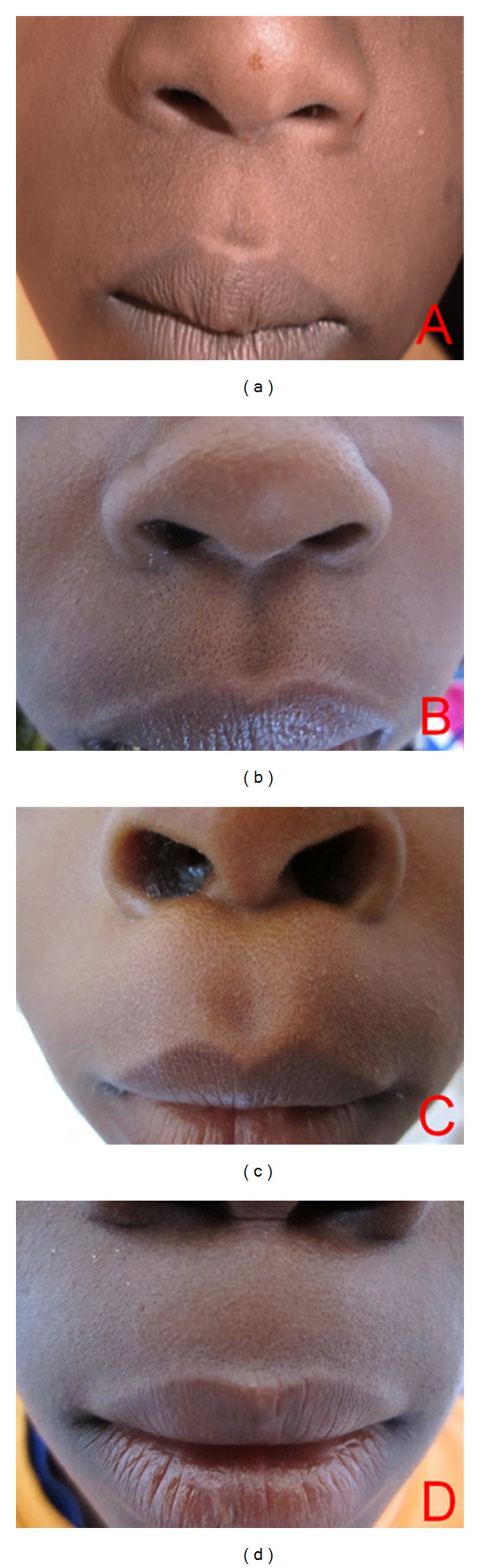
Pictures of philtral shape classification [13]. (a) Triangular type, (b) parallel type, (c) concave type, and (d) flat type.

**Figure 4:**
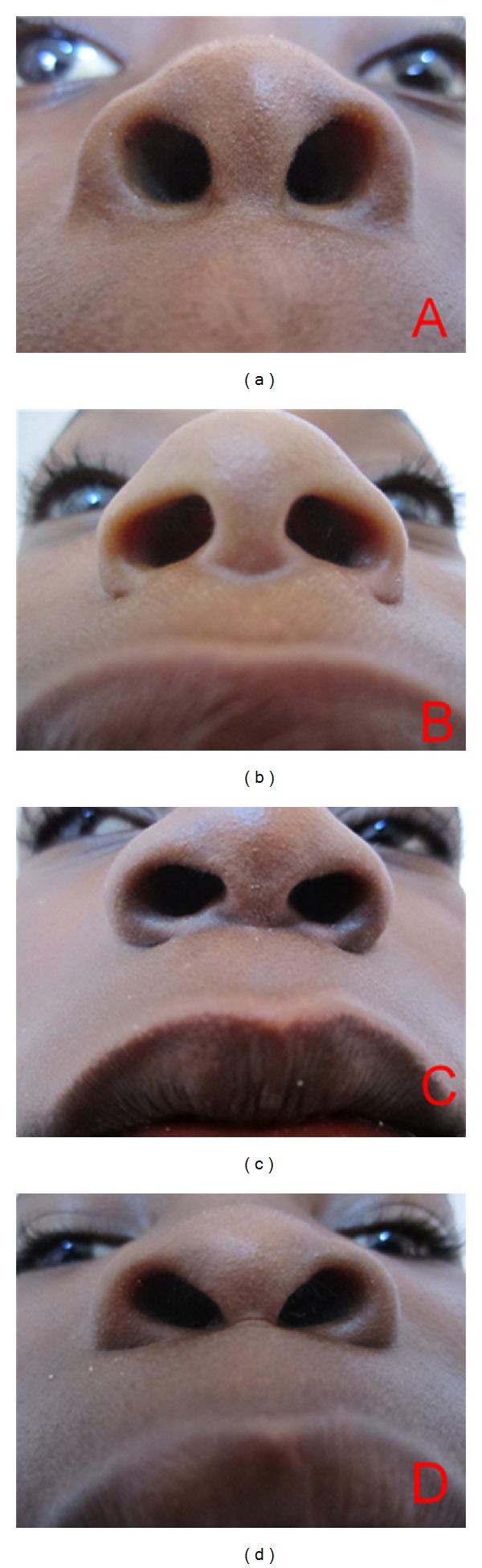
Pictures of nostril shape classification. (a) Round type, (b) rectangular type, (c) teardrop type, and (d) triangular type.

 Photographs of the philtral columns and nostril shape were obtained by the primary author (A.I.). Photographs were taken with a Canon IXUS 130 digital camera, 14.1 megapixels. (Canon United Kingdom). We took pictures of the philtrum and the upper lip in frontal view. The children had their head bent back, vertically exposing the nostrils. From this position, photographs of the nostril shape were obtained. We selected four photographs each, of the shape of the philtral columns and nostrils (Figures 3 and 4). The photographs were then placed onto two PowerPoint slides for viewing. We subsequently recruited 60 volunteers (30 professionals and 30 laypersons) to evaluate the photographs. The professionals consisted of consultant plastic surgeons, surgery residents, and nurses. The laypersons included members of the office staff of the department of surgery, friends, and relatives of patients at the surgical outpatient department. Each volunteer was asked to rank the photographs of the shape of the philtral column and nostril shape based on their aesthetic preference. They were directed to rate each on a scale from 1 to 5, with 1 representing “very unattractive” and 5 as “very attractive.”

On the basis of age, volunteers were divided into 2 groups: 35 years or older and younger than 35 years. Using Mann-Whitney tests, statistical comparisons based on volunteer age, sex, and profession were completed. *P* < 0.05 was considered as statistically significant. 

## 3. Results

### 3.1. Morphology of Philtral Column and Nostrils

In both genders, the triangular type was the most common, and the parallel type was the second most common. The distribution of participants into groups regarding sex was not significantly different in the triangular group compared with the parallel, concave, and flat groups (*P* = 0.207). The result of the classification of the shape of the philtral column is shown in Table 1. 

The teardrop-shaped nostrils were more common than the other types (Table 2). Triangular shaped nostril was the commonest in male while the teardrop shaped nostril was the commonest in females. This distribution was, however, not statistically significant, *P* = 0.690.

### 3.2. Aesthetic Preferences of Philtral Column and Nostril Shape

The professionals ranked the flat and parallel philtral columns higher while the laypersons scored the triangular and concave philtral columns more favorably. However, these differences are not statistically significant (Table 3). The distinction in the rankings between female and male volunteers was also less clear. Compared with females, males prefer the parallel and triangular shaped philtral column, whereas females ranked the flat and concave philtral shaped columns higher. This trend was also not statistically significant. The only statistically significant ranking was based on volunteer age, with volunteers less than 35 years preferring the concave shaped philtral column. 

For nostril shape, the mean scores the professionals were slightly higher than the laypersons (Table 4). The mean scores for the professionals ranged 28.25–35.35 while the mean scores for the laypersons ranged 25.65–32.75. The aesthetic ranking of the triangular shaped nostril was statistically significant. *P* = 0.025 between the two groups. Comparisons of rating scores between male and female volunteers revealed higher mean scores by females (Females 30.56–31.08 versus Males 29.88–30.43). This distribution did not show a statistically significant difference. When comparing the aesthetic ratings of volunteers less than 35 years and those older than 35 years, the mean scores in the triangular and round shaped nostrils were statistically significant (*P* = 0.015 and *P* = 0.025).

## 4. Discussion

Objective evaluation of the face is based on measurements, proportions, and shapes. A great body of work in facial anthropometry is that of Farkas, who established a database of norms that are well accepted as linear, angular, and surface contour reference values [9, 17]. Other reference values are from 2D cephalometric and photographic assessments [13, 18–21]. More recently, the laser scanning technique, the contact-type 3-dimensional measurement technique using facial plaster models, and the measurement technique using 3-dimensional computed tomography are considered to be able to 3- dimensionally measure the complicated shape of the face and to produce images on a computer screen [13, 20, 22]. 

The analysis obtained in this study was based on two-dimensional basal views and photographs of the philtral columns and the nostrils because they were economical, convenient, and noninvasive. Photographs were chosen to assess aesthetic preferences because they were proven to be reliable in previous studies [23, 24]. The strengths and limitations of photographic assessments must be appreciated. It is sensitive to the angle from which the photograph is taken and the position of the head. Because photographs are taken from varying distances with lenses of different focal lengths, the magnification of the final image is unknown. Therefore, it is unsuitable for absolute measurements, unless standardized procedures are followed to ensure a consistent, known magnification. However, it is ideally suited to the evaluation of proportions and shapes, as the magnification factor is eliminated [15].

The morphology of the philtral columns was classified into four types for Japanese children by Mori et al. in 2005 [13]. Amongst Nigerian children, in both genders, the triangular type was the most common, and the parallel type was the second most common. This contrasts the findings in Japanese children where the parallel type was the most common in both genders, and the triangular type was the second most common [13]. This corroborates the distinct differences in facial morphology further emphasizing the need for separate standards for facial analysis.

The characteristics and differences of the shapes of the nostrils have been studied in several racial groups [13–15, 18, 21, 25, 26]. Among the classifications of nostrils, Farkas' classification divides the shape of nostrils by the angle between the right and left long nostril axes [25]. We used 4 main nostril forms based on Farkas' classification in an earlier report [15] and replaced the heart shaped nostril with the rectangular shaped nostril in our classification. Our data was compared with Ofodile's data in African Americans [26], and the teardrop nostril was the commonest in both studies. However, the African American group also had a type III in females which accounted for 4%. The type III nostril shape was not present in this study. 

Although the trend in this study demonstrates that female volunteers prefer the concave philtrum and triangular shaped nostril compared with male volunteers, these differences were not statistically significant. Furthermore, no differences were seen between professionals and laypersons volunteers for philtral column shape. Although some volunteers in this study were friends and relatives of patients in the hospital, most were graduate school students, a factor which introduces bias based on advanced educational level and urban residence [16]. In this study, it was found that the nostril shapes had higher aesthetic scores compared to the philtral columns shape. Two explanations for this finding are possible. First, the subjects were asked to not smile for an accurate assessment of philtral column shape. Rating pleasant, smiling lips may have resulted in higher aesthetic scores; however, this would have come with a price of decreased accuracy in philtral column assessment with the introduction of teeth and altered labial proportions. Another reason is that perhaps the ratings on photographs that are cropped are generally not a skill familiar to the volunteers and it may influence the results [27]. The difference in rating demonstrates the variance in what is perceived as attractive. Aesthetic judgments are subjective and may vary over time. It is therefore difficult to measure. Clinicians should thus develop patient-centered treatment goals through awareness of the aesthetic preference of their society [28, 29]. The acknowledgment of this is increasingly important in the modern era because our society continues to become more heterogeneous [16].

### 4.1. Clinical Correlates

Cleft lip and palate is one of the most common deformities of the upper lip-nose subunit in Nigeria [30]. One of the major goals of surgery is to improve the aesthetic appearance of the face and thereby improve the patient's social acceptability [4, 19, 31, 32]. Unfortunately, the nature of the unilateral and bilateral cleft lip and nasal deformity makes the asymmetry difficult to correct completely [5, 6, 27, 33–36]. A considerable number of children following cleft lip/nose repair in our institution had triangular shaped nostrils while others had heart shaped nostrils. Heart shaped nostrils are conventionally considered to be a typical nostril shape following cleft lip surgeries [13]. We also observed that the reconstruction of the cleft lip deformity (unilateral and bilateral) using modification of Millard's rotation advancement technique resulted in a parallel shape philtral column. A key feature of humans is bilateral symmetry and the human eye is sensitive to differences in the two sides of paired structures [36]. Strict observation of hard and fast rules should, however, not blind us to the subtle uncertainty expressed by Picasso “art is not the appreciation of a canon of beauty but what instinct and the brain can conceive of outside the canon” [37]. Cleft lip surgeons must thus continue to be perfectionists and be willing to work in fractions of millimeters for the best possible results [9, 38].

## 5. Conclusion

Reconstruction of the upper lip and nostrils requires a thoughtful combination of art and science. We have contributed towards the science by describing the morphology of the philtral columns and nostrils and the aesthetic preferences in Nigerian children. We found that the triangular shaped philtral column is the commonest in boys and girls. There are significant age differences in the aesthetic rankings of philtral columns and nostril shapes. However, the aesthetic preferences are similar for professional status and gender. We hope that the results of our study and reconstructive surgery will intersect at a point to treat disfigurements of the upper lip-nose subunit in Nigerian children successfully. 

## Figures and Tables

**Table 1:** The distribution of participants related to their sex and shape of philtral column.

Shape of philtrum	Total	Male		Female	
	*N*	*N*	%	*N*	%
Parallel	19	8	42.1	11	57.9
Triangular	55	34	61.8	21	38.2
Concave	12	4	33.3	8	66.7
Flat	14	8	57.1	6	42.9

Total	100	54		46	100

**Table 2:** The distribution of participants related to their sex and shape of the nostrils.

Shape of nostril	Total	Male		Female	
	*N*	*N*	%	*N*	%
Triangular	39	23	59	16	41
Round	13	7	53.8	6	46.2
Teardrop	42	22	52.4	20	47.6
Rectangular	6	2	33.3	4	66.7

Total	100	54		46	

**Table 3:** Mean scores of philtral column shape preferences based on volunteer professional status, sex, and age.

Philtral column shape	Professional status		*P* value	Sex		*P *value	Age		*P* value
	P^⋆^	L^⋆^		M	F		<35	>35	
Flat	32.40	28.60	0.362	29.57	31.37	0.666	28.73	33.79	0.247
Parallel	32.67	28.33	0.320	31.76	29.32	0.577	27.73	35.64	0.083
Triangular	30.38	30.62	0.957	31.40	29.66	0.691	30.29	30.88	0.898
Concave	27.40	33.60	0.153	28.17	32.68	0.299	33.95	24.10	0.030

P^⋆^: professional; L^⋆^: layperson.

**Table 4:** Mean scores of nostril shape preferences based on volunteer professional status, sex, and age.

Nostril shape	Professional status		*P* value	Sex		*P* value	Age		*P* value
	P^⋆^	L^⋆^		M	F		<35	>35	
Triangular	35.35	25.65	0.025	29.88	31.08	0.782	26.64	37.67	0.015
Rectangular	30.38	30.62	0.956	30.43	30.56	0.975	30.13	31.19	0.809
Tear drop	30.48	30.52	0.994	30.22	30.76	0.902	31.08	29.43	0.718
Round	28.25	32.75	0.308	29.97	31.00	0.815	34.13	23.76	0.025

P^⋆^: professional; L^⋆^: layperson.
